# Health and social support services to HIV/AIDS infected individuals in Tanzania: employees and employers perceptions

**DOI:** 10.1186/1471-2458-14-630

**Published:** 2014-06-20

**Authors:** Telemu Kassile, Honest Anicetus, Raphael Kukula, Bruno P Mmbando

**Affiliations:** 1 Faculty of Science, Sokoine University of Agriculture, P.O Box 3038, Morogoro, Tanzania; 2 Directorate of Preventive Services, Ministry of Health and Social Welfare, P.O. Box 9083, Dar es Salaam, Tanzania; 3 Occupational Health and Safety, P.O. Box 790055, Dar es Salaam, Tanzania; 4 National Institute for Medical Research, Tanga Research Centre, P. O. Box 5004, Tanga, Tanzania

**Keywords:** Family member, Treatment and nutritional support, Workload, Workplaces

## Abstract

**Background:**

HIV is a major public health problem in the world, especially in sub-Saharan Africa. It often leads to loss of productive labour and disruption of existing social support system which results in deterioration of population health. This poses a great challenge to infected people in meeting their essential goods and services. This paper examines health and social support services provided by employers to HIV/AIDS infected employees in Tanzania.

**Methods:**

This was a cross-sectional study, which employed qualitative and quantitative methods in data collection and analysis. Structured questionnaires and in-depth interviews were used to assess the health and social support services provision at employers and employees perspectives. The study participants were employees and employers from public and private organizations.

**Results:**

A total of 181 employees and 23 employers from 23 workplaces aged between 18–68 years were involved. The results show that 23.8% (i.e., 20.4% males and 27.3% females) of the employees had at least one member of the family or close relatives living with HIV at the time of the study. Fifty six percent of the infected employees reported to have been receiving health or social support from their employers. Employees’ responses were consistent with those reported by their employers. A total of 12(52.2%) and 11(47.8%) employers reported to have been providing health and social supports respectively. Female employees (58.3%) from the private sector (60.0%) were more likely to receive supports than male employees (52.6%) and than those from the public sector (46.2%). The most common health and social support received by the employees were treatment, and nutritional support and reduction of workload, respectively.

**Conclusions:**

HIV/AIDS infected employees named treatment and nutritional support, and soft loans and reduced workload respectively, as the most important health and social supports they needed from their employers. This study provides baseline information for further studies on provision of health and social support services by employers to HIV/AIDS infected employees in the context of a developing economy like Tanzania.

## Background

HIV remains a major public health affliction in the world, particularly in sub-Saharan Africa (SSA). Of the estimated 34 million people who were living with HIV worldwide by the end of 2011, 23.5 million were from SSA. Also, in 2011, SSA accounted for about 72% of 2.5 million new HIV infections. Meanwhile, reports show that 4.9% of adults in SSA were living with HIV as opposed to 1.0% of adults in the Caribbean, Eastern Europe and Central Asia [1].

In Tanzania, UNAIDS report [1] indicated that by the end of 2011, approximately 1.6 million people were living with HIV and 1.3 million of them were aged 15 years and above. Other findings from the Tanzania HIV/AIDS and Malaria Indicator Survey [2] show that in Mainland Tanzania, HIV prevalence for the economically active population (15–49 years) was 5.3% and the most infected individuals were females, residing in urban areas.

In endemic countries, the HIV/AIDS epidemic affects the community differently, since infected persons can be a family member, friend, neighbour, or a workmate [3]. The epidemic has negative consequences on the livelihood of the people, including loss of productive labour-force and income, decrease in food reserves, as well as disruption of the existing social support systems [4]. The impact of all these is poor nutrition and health status of HIV infected individuals. Moreover, as an income of the household declines, the household concerned is forced to adopt coping strategies. Some of such strategies may have impact on the general livelihood of the household, including exacerbating poverty [5], and whose consequence is limited access to social services including health care [6].

Low income countries have made considerable progress in improving the performance of health and community services. Despite this development, people living with HIV/AIDS still experience relentless social and cultural barriers in accessing health and community services. Such barriers are related to restricted social support, worry of disclosure, interpersonal aggression, and stigma [7,8]. For example, a review study by Musheke and colleagues [8] found the availability of treatment and social support services to be a key determining factor in the individuals’ decision to undertake HIV testing.

Despite the availability of information on the impact of HIV/IADS in the workplaces as well as guidelines for mitigating the epidemic and its consequences [4], little is known on the roles played by different employers and employees in fighting the epidemic. The available evidence shows that employers recognize that HIV/AIDS put their workers and their families at peril. The impact of HIV/AIDS on the affected parties may take different forms, including job satisfaction and performance, stress, and relationship of the employee with fellow workers. Meanwhile, companies fail to harness the full potential of their employees by the time such employees are suffering illnesses relating to AIDS. These issues may have huge influence on the employee’s subsequent course of action, which may include decisions to quit the job [9].

In Tanzania, the National Policy on HIV/AIDS [6] acknowledges that high costs of care for people living with HIV/AIDS places a huge burden to the already overburdened households, thus persuading all members of the community to actively participate in the prevention and control of the HIV/AIDS pandemic.

This paper explores availability, extent, and types of health and social support services, which employers extend to HIV/AIDS infected employees in Tanzania.

## Methods

### Study design

This involved a cross-sectional study design, which utilized quantitative and qualitative techniques. The study adopted the convergent mixed methods design in which quantitative and qualitative data were collected simultaneously, but analyzed separately. The study involved populations of workers in workplaces in the cities of Dar es Salaam, Arusha, and Tanga. Arusha and Tanga regions are located in the north-eastern part of Tanzania with populations of 1,694,310 and 2,045,205 respectively while Dar es Salaam region is located in the eastern part of the country and has the population of 4,364,541 [10].

From each of the three cities, workplaces were selected primarily based on the nature of activities undertaken, but focusing on high chances of meeting the target respondents for interviews. Activities, which were considered desirable, were those for which most of the employees were expected to be on permanent employment. These included health-related activities, and food and beverages, among others. Moreover, workplaces with limited access, that is, workplaces (such as armed forces), which are usually intricate in terms of granting permission to conduct research activities, were also excluded. In total, twenty-three workplaces from public and private sectors were selected.

### Study population

The primary respondents for the study were employees of the selected workplaces. In addition, workplace managers, herein referred to as *employers,* were also involved in the study in order to have their opinions as supervisors of the employees. One employer or a delegate from each workplace was included. Meanwhile, in this study, an infected employee was defined as an employee either living with HIV/AIDS or having a member of the family or a close relative living with HIV/AIDS or both.

### Sampling procedure

Casual labourers and part-time employees were considered ineligible for the study. The study considered first, that all eligible employees at each workplace had equal chance to participate in the study. Secondly, a few employees from each workplace were reasonable to provide the status quo of the provision of health and social support services by employers to HIV/AIDS infected employees. For these reasons, at each workplace, the study selected between 4–10 employees for interviews. Simple random sampling technique was used to select required employees from each workplace.

### Data collection

The data for the study were collected using structured questionnaires. These were administered to employees and employers and consisted of items developed based on a review of both local [11] and global [12] literature on HIV/AIDS, particularly in workplaces. The questionnaire for employees consisted of 29 items while that of employers had 31 items. Besides the questionnaires, an interview guide was also used to collect opinions from the employees. The guide had less structured questions, allowing flexibility in interviewing as a way of maximizing responses. Data collection took place for about two weeks in March 2006. The interviewers had one-day training on various aspects of the study. Data collection process was done concurrently in all the study areas. The following are some of the issues that featured in the questionnaires.

#### Employees

The questionnaire (Additional file 1) for employees collected two types of information. First, employees’ socio-demographic characteristics such as age, sex, marital status, education, whether or not the respondent had a child, and time (in years) the respondent had been employed in his/her current job. The second type of information was on specific aspects of the study, including pre-employment HIV test and whether the test was a prerequisite for employment, and whether the respondent had a member of the family living with HIV. Other types of information collected were whether the respondent received any health or social support from his/her employer, the types of health and social support provided by the employer, and specific health and social support that the employee needed from his/her employer.

#### Employers

Besides general information about the workplace, the questionnaire for employers (Additional file 2) sought for specific information on whether the workplaces had any employee living with HIV/AIDS and the mechanism used to ascertain this. Other items sought to collect information on whether or not HIV pre-employment test was a requirement at the workplace; whether or not the workplaces provided any health or social support to HIV/AIDS infected employees; and the types of support provided. Each workplace had only one response questionnaire administered for employer.

The questionnaires for both employees and employers were written in English first and later translated into Kiswahili (the language, which is widely spoken in Tanzania). This was purposely done in order to facilitate communication during the data collection process. Later on, the Kiswahili versions were translated back to English to ensure the original meanings were maintained. The final versions were pre-tested in order to determine suitability of the questionnaires’ items. The interviews were conducted in Kiswahili.

#### Focus group discussions (FGDs)

In addition to the questionnaires, FGDs of 4–10 employees not initially involved in the interviews were also conducted with a view to first, gain more insights on key aspects of the study and secondly, to triangulate the responses from the two other aforementioned categories of respondents. Participants for FGDs were selected purposefully with the help of employers in the respective workplaces in the study areas. One FGD was conducted at each workplace, thus making a total of 23 FGDs. Guiding discussion questions (Additional file 3) included for example, what measures are taken by the employers to handle cases of employees with long term illnesses? Does the employer provide any health or social support services to HIV/AIDS infected employees? Each FGD session had two facilitators and a note taker. The process involved introduction of the research team and participants. This was succeeded by an account of the objectives of the research. Overall, the qualitative part of the study adhered to RATS guidelines on qualitative research.

### Ethical consideration

Ethical clearance was obtained from the Ministry of Health and Social Welfare. Then, permission to conduct the study was obtained from the Municipal Councils in the respective cities and from the management of each of the selected workplaces. In addition, written consents were obtained from each respondent or participant prior to the interviews or discussions respectively.

### Statistical analysis

Numerical descriptive statistics were mainly employed to summarize the data and were carried out in the SAS System for Windows version 9.2. The arithmetic mean and standard deviation (SD), and frequency along with percentage (%) were computed for continuous and discrete variables respectively. The Kruskal-Wallis rank sum and the chi-square tests were employed to check for differences between groups for continuous and skewed, and for categorical variables respectively. Moreover, for skewed variables, the median was used instead of the mean to describe the characteristics of the data. Qualitative data were analysed mainly through identification of themes or patterns of phrases or texts recorded. This was achieved through reading and re-reading the texts and identifying coherent categories that summarize and bring meaning to the text.

## Results

### Characteristics of workplaces

Twenty-three workplaces were selected for the study and were evenly distributed across the study areas: 8 (34.8%), 7 (30.4%), and 8 (34.8%) were from Arusha, Dar es Salaam, and Tanga cities respectively. The median number of employees for workplaces in Arusha, Dar es Salaam, and Tanga were 95, 166, and 92 respectively. Most of the workplaces 11 (47.8%) were industries (pharmaceutical, textile, cement, foods and beverages, and construction), while 4 (17.4%) were health facilities, 3 (13.0%) were farms and 13.0% comprised of a municipal council, construction companies and a hotel. Over 82.6% (*n* = 19) of the workplaces were privately owned and the remaining, 17.4% (*n* = 4) were publicly owned.

### Characteristics of the study population

Table 1 provides background information about the study population. A total of 181 employees, 93 (51.4%) males participated in the study. There was a significant association between the study area and the sex of the respondents (*p* <0.001). The majority of respondents, 66 (36.5%) were from Tanga. The age of participants ranged from 18–68 years (mean = 36.5; SD = 9.5). The average duration of employment was 7.8 (7.5) years. Employees in Tanga city had the highest duration in their workplaces while Dar es Salaam had the least.

**Table 1:** General characteristics of the study population

	**Study area**				***P*-value**
**Characteristic**	**Arusha**	**Dar es Salaam**	**Tanga**	**Total**	
Sex, *n* (%)					
Male	17 (29.8)	40 (69.0)	36 (54.5)	93 (51.4)	<0.001
Female	40 (70.2)	18 (31.0)	30 (45.5)	88 (48.6)	
Age in years, mean (SD)	34.7 (7.9)	36.2 (9.0)	38.4 (10.9)	36.5 (9.5)	0.244
Years at workplace, mean (SD)	7.3 (6.8)	5.5 (6.3)	10.4 (8.3)	7.8 (7.5)	<0.001
Education, *n* (%)					
Primary or less	29 (50.9)	7 (12.1)	43 (65.2)	79 (43.6)	<0.001
Technical	0 (0.0)	4 (6.9)	8 (12.1)	12 (6.6)	
Secondary and above	28 (49.1)	47 (81.0)	15 (22.7)	90 (49.7)	
Marital status, *n* (%)					
Single	17 (29.8)	19 (32.8)	16 (24.2)	52 (28.7)	0.565
Married	40 (70.2)	39 (67.2)	50 (75.8)	129 (71.3)	
Ever had a child, *n* (%)					
Yes	46 (80.7)	42 (72.4)	56 (84.8)	144 (79.6)	0.223
No	11 (19.3)	16 (27.6)	10 (15.2)	37 (20.4)	

The majority of the respondents, 79 (43. 6%) had primary or less education and the number was higher, 43 (65.2%) among the employees in Tanga city than other cities. The proportion of females with education level of secondary and above was higher (*n* = 48, 54.5%) than that of males (*n* = 42, 45.2%). However, the difference was not statistically significant, χ^2^ = 3.581, *p* = 0.167. Moreover, most of the educated respondents, 47 (81.0%), were working in Dar es Salaam, while the least, 43 (65.2%), were working in Tanga. Most of the respondents, 129 (71.3%), were married and more than three-quarter, 144 (79.6%), had at least one child (Table 1).

### Pre-employment HIV test

A total of 25 (13.8%) of the study population reported that they had undergone a pre-employment HIV testing in connection to their occupation. However, the rate did not vary significantly between males, 12 (12.9%) and females, 13 (14.7%). In addition, 56.0% of the participants who had undertaken the test indicated that HIV status was one of the requirements for their employment. Female employees were more likely to report about the testing requirement in their workplaces, 9 (69.2%) than their male counterparts, 5 (41.7%). Also, participants in privately owned workplaces were more likely to report HIV testing requirement than was the case in the public sector: 2 out of 5 (60.0%) and 12 out 20 (40.0%) respectively. Furthermore, the findings show that statistically, the nature of activities at the workplaces was significantly associated with the requirement of undergoing pre-employment HIV test. In particular, the employees working in hotel, 6 (85.7%) reported about the existence of this requirement in their workplace than those in other lines of business, including those working in the construction sector, 1 (8.3%) or in the farms, 2 (8.3%) (*p <* 0.001).

A total of 135 (74.6%) participants were of the opinion that once a person is infected with HIV, he/she should disclose the status to the employer. Only 35 (19.3%) individuals stated that they should keep their status private, while 11 (6.1%) did not know what one should do if infected with HIV. Female respondents, 16 (18.2%) were less likely to report that the HIV infection status should be confidential as compared to male respondents, 19 (20.4%). However, the difference was statistically not significant (*p* = 0.871). Also, there was no significant association between the type of workplace (public or private) and readiness to disclose HIV status to the employer (*p* = 0.206). The participants from Dar es Salaam reported that the individual should keep it private as indicated by 16 respondents (27.6%) as compared to those from Arusha as indicated by 5 respondents (8.8%) or from Tanga, 14 (21.2%). The observed difference was statistically significant (*p* = 0.006). It was further revealed that 43% (*n* = 10) of the workplaces had health education programmes for employees and their families on matters related to HIV/AIDS. The programmes were available in all public workplaces and in 31.6% (*n* = 6) of the privately owned workplaces (*p* = 0.012). There was no association between availability of health education programmes in the workplaces and the cities surveyed in the study (*p* > 0.05).

### HIV/AIDS infected employees

A total of 43 (23.8%) of the participants reported to have at least one family member or close relatives living with HIV or AIDS at the time of the study. This aspect was reported more frequently by females, 24 (27.3%) than was by males 19 (20.4%), however, the difference was not significant (*p* = 0.280). Also, there was no association between reporting presence of an HIV infected family member or close relative and study regions (*p* = 0.236). The nature of the responses from 23 employers was consistent with those of employees. A little more than one-third (*n* = 8, 34.8%) of the employers reported to have been aware that some of their employees were living with HIV.

Irrespective of whether or not the employer had an infected employee, all employers were asked to specify the mechanisms they have been using to identify the employees who were infected with HIV/AIDS. Several mechanisms stated were: doctors’ prescription 5 (21.7%), specific unit that dealt with HIV/AIDS matters at the workplaces 2 (8.7%), and self-declaration by individual employees 1 (4.3%). Other mechanisms mentioned include hearsay/ rumours, 6 (26.1%), as well as a combination of rumours and self-declaration by the individual employees, 2 (8.7%).

#### Support to HIV/AIDS infected employees

**Figure 1:**
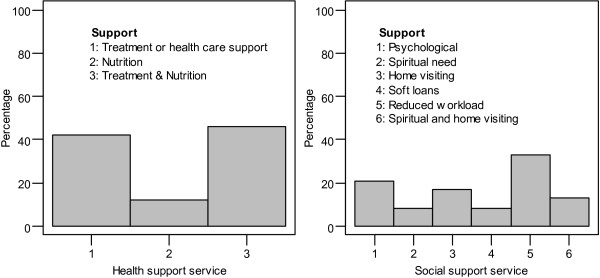
Health and social support services received by employees from employers.

More than half (*n* = 24, 56%) of the HIV/AIDS infected employees reported to have been receiving health and social supports from their employers. Female employees, 14 (58.3%) and those in the private sector, 18 (60.0%) more frequently indicated to receive supports from their employers than male employees, 10 (52.6%) and those in the public sector, 6 (46.2%). Figure 1 provides types and frequency of health and social supports provided by employers to their respective HIV infected employees. Most common health support provided included treatment and nutrition (45.8%) while on social support; reduced workload was the mostly reported support (33.3%).

The reported responses of employers with respect to health support services received by employees were largely consistent with responses given by the employees. A total of 12 (52.2%) and 11 (47.8%) employers reported to have been providing health and social supports respectively. Treatment or health care was the main reported health support service provided by the employers. On social support, psychological, non-discriminatory conduct of the employees at the workplace, and a combination of psychological and income generating activities were the most common social support services provided by the employers (Table 2).

**Table 2:** Health and social support provided by employers to HIV/AIDS infected employees

**Health support (*n*** **= 12)**	***n *** **(%)**	**Social support (*n*** **= 11)**	***n *** **(%)**
Treatment or health care support	12 (100)	Psychological	3 (27.3)
		Non-discriminatory attitude	3 (27.3)
		Psychosocial and income generating activities	2 (18.2)
		Psychological and spiritual needs	3 (27.3)

With regard to provision of treatment to infected personnel and their families, results show that more than two-thirds, (*n* = 16, 69.6%) of the employers reported to have treatment provisions for workers and their families. Of those who reported to have treatment provisions, 7 (43.8%) said that their employees were treated at a health facility available at their workplaces and 4 (25.0%) reported that their employees were treated at specific health centres identified by the management. Some, 3 (18.8%) of the employers cited health insurance schemes such as the National Health Insurance Fund as a mechanism of rendering health care services to their workers and their families at their workplaces. The remaining, 2 (12.5%) of the employers reported to have treatment provisions for their workers and their families only for minor complaints such as typhoid and other related food borne diseases as agreed between the company/organization and employees.

### Specific support required

Respondents in the study (irrespective of their status, whether infected or not) were all asked to provide suggestion on specific health or social support, which they thought would be more helpful to HIV/AIDS infected employees. The findings show that, on health support, treatment and nutritional support was the leading recommendation, while on social support soft loans and reduction of workload were the highly recommended (Table 3). Overall, almost all, 176 (97.2%) participants supported the notion that employers should provide support to HIV/AIDS infected employees.

**Table 3:** Specific health and social support services recommended by employees

**Health support (*n*** **= 181)**	***n *** **(%)**	**Social support (*n*** **= 181)**	***n *** **(%)**
Treatment	64 (35.4)	Psychological	18 (9.9)
Nutritional support	4 (2.2)	Spiritual needs	11 (6.1)
Treatment and nutritional support	93 (51.4)	Soft loans	36 (19.9)
Don’t know	20 (11.0)	Reduced workload	4 (2.2)
		Soft loans and reduced workload	80 (44.2)
		Income generating activities	2 (1.1)
		Don’t know	30 (16.6)

Discussions held between the researchers and employees on different aspects of particular interest in the study during FGDs revealed similar responses across cities in most of the aspects. On the question of measures taken against people with long illnesses, majority of the study participants were of the opinion that there were no any measures. Where the measures existed; they were meant for preparation of termination of employment. For example in six of the workplaces participants said: “e*mployees with long illnesses are normally granted three months sick leave with half-monthly salary. After these three months, the employee is terminated on medical grounds and given his/her full terminal benefit”*. In another workplace employees felt that there were no stipulated guidelines, but rather some employees could receive the services on friendly basis or as an offer. A participant said: “*No clearly stipulated directive on what employees have to do on this issue, some employees are given support not as their right but as friends or relative of employer* (meaning that nepotism was accorded priority)*”.* The participants in this working place went further and suggested that having HIV is not the end of someone’s working capacity, so an employer should provide timely support to enable recovery. Here is a quote from one of the participants: *“the employer should help employees to get timely treatment and diet as well as giving them adequate time to recover after starting treatment. An infected person may recover and live for ten or more years, thus he/she can continue being productive to his/her employer”.*

The opinions of the employees were largely consistent with the views given by the employers. It was revealed in the study that among the measures taken by employers against workers with long illnesses include giving long sick leave, 7 (30.4%), paying all medical expenses, 7 (30.4%), or terminating employment, 5 (21.7%).

With regard to provision of health and social support services to HIV/AIDS infected employees, participants in most of the workplaces were of the opinions that the services were usually not provided. In the workplaces where the services were provided, participants disclosed that there was no clearly defined or known system of provision of the services. In some workplaces, the services were for few employees. For example, in five of the workplaces, some participants were concerned with shortage of basic support from the employers to people infected with HIV. It was said: “*Few of the infected employees get health support through health insurance schemes”*. Also, in some workplaces, health and social support services were provided for higher-level employees. In such workplaces, some participants informed of existence of incidences of lower-level HIV/AIDS infected employees been given less care, but when the employees died, the employer provided all burial costs. From the discussions, the study revealed that general health care and few social support services like psychological were provided. Some participants expressed that: “*General health care is provided but not special treatment for HIV/AIDS infected employees and incentives like cement, good performance awards, Christmas bonus, etc., are provided*”.

## Discussion

### Pre-employment HIV test

This study has revealed that some employees undertook a pre-employment HIV test as one of the conditions for their employment. This finding is consistent with the observations reported elsewhere in Tanzania that some institutions in both public and private sectors had developed and adopted guidelines, which included pre-employment testing [13]. Tanzania’s National Policy on HIV/AIDS of 2001 [6] proscribes discrimination against people living with HIV/AIDS on matters pertaining to employment and/or social services. In this regard, pre-employment HIV screening and direct or indirect screening of those already employed is considered by the Policy to be unlawful. Of particular interest in this respect is the finding that a significant proportion of the participants were of the view that once employees know their HIV status, such employees should then disclose their HIV/AIDS status to their employer. However, this could only be feasible if the infected employee is well informed of the advantages and disadvantages of his/her decision to disclose his/her HIV status to the employer and that, the former outweighs the latter.

The findings show that at the time of the study, about 24% of the workers in the study areas had a family member or a close relative infected with HIV. This proportion (24%) though may not be a true representation of the proportion of the infected workers in the study, and as it seems, it is consistent with the earlier noted observation that HIV/AIDS affects everyone in some way [3]. Moreover, the finding that female employees were more likely to report having a family member living with HIV could reflect women’s active participation in the production of non-market goods such as health and nutrition in the context of developing countries as long noted by Khandker [14]. The latter inference is consistent with the finding from the current study that female employees were less likely than males to perceive that if one knows his/her status he/she should not keep it private instead inform the employer. Differential rates in reporting between females and males could also be a reflection of differences in susceptibility to the pandemic between them as earlier noted [2].

#### Health and social support services

The study has established that more than half of the HIV/AIDS affected employees received both health and social supports from their employers. Both treatment and nutritional and reduced workload accounted for most of the health and social supports received by the employees from their employers. However, the system of provision of these services was considered by some of the employees as not explicit. Some participants during FGDs were of the view that the health and social support services were not known because of privacy which some of the infected employees wanted to maintain regarding their HIV/AIDS status. This poses a great challenge in the management of HIV/AIDS in the workplace. Jantjie [9] argues that without knowing the HIV prevalence of an organisation, the efforts of implementing HIV prevention and control measures at the workplace would be compromised, and indeed the present study reveals this view as being relevant. In an attempt to address HIV/AIDS in the workplace, policies, which call upon employers to be involved in the fight against the epidemic, have been proposed in different settings. For example, Cone [15] proposes a policy that aim at dealing with HIV/AIDS in the workplace through case management and provision of services such as accommodation to employees living with AIDS, among others. According to UNAIDS and the International Organisation of Employers [16], days of work lost due to HIV/AIDS-related illnesses is one of the effects of the epidemic at the micro-level. The direct consequence of this impact on the individual companies is decreased productivity. As suggested by some participants in the study, provision of services such as treatment and nutritional could help the infected employee regain his/her work ability thus minimizes repeated absenteeism from work.

The need for social and health support services among the infected employees from their employers as revealed in the present study is largely reflected by the information on level of education of the employees. Most of the employees had primary or less education, which signifies that overall, most of them were less skilled and thus performed manual related jobs instead of sophisticated posts that required high-level skills. This is an indication that they are not well paid, as a result, they cannot afford to pay for basic needs required among HIV infected individuals, hence the need for support services such as soft loans, nutritional, and health care from their employers. This observation is consistent with evidence in the microeconomic literature. For example, Birdsall and colleagues [17] and Ross and Wu [18] reveal that highly educated individuals receive higher wages, and hence they are more capable in managing the health and nutritional status of their families.

Despite the potential contribution of the present study on the subject of HIV/AIDS in the workplace, the study had a number of limitations that are worth mentioning. The study did not involve a detailed design to permit identification of key variables that influence and hinder provision of health and social support services by employers to the infected employees. In this connection, information such as income or expenditure and demographic structure of the infected employees would have provided more insights into the subject matter. Also, the study considered a sample size of 181 with only 4–10 employees from each workplace. Although the sample was selected on random basis, it might not have captured the aspect of representativeness, thus the results may not be generalized to the population of workplaces in Tanzania. Moreover, the selection of the workplaces did not consider homogeneity of the workplaces. Probably because of this limitation, educational achievement of the employees varied between cities. This imbalance makes the interpretation of the impact of educational achievement on the demand for health and social support services from employers difficult. Another limitation is related to the time elapsed since the study was carried out. As revealed above, the study was conducted in 2006. Therefore, it is most likely that some of the results in the current study may not reflect the existing situation of HIV/AIDS in the workplace in Tanzania, particularly with reference to HIV pre-employment testing.

## Conclusion

This study provides baseline information for further studies on the provision of health and social support services by employers to HIV/AIDS infected employees in the context of a developing economy like Tanzania. It has established that the provision of health and social support services to HIV/AIDS infected employees is a notion that requires particular attention since still the employees are missing some of the important rights and opportunities of being treated as eligible workers in various aspects. Although almost all respondents supported the idea that employers should provide support to HIV/AIDS infected employees, the support provided seemed to be inadequate since a little above half of the total infected employees received support from their employers. It is surprising that there being no specific system of supporting HIV/AIDS infected employees in the workplaces and this means that the health system and employers in both sectors have substandard or un-uniform modalities of operation in this area.

## Abbreviations

AIDS: Acquired immunodeficiency syndrome; FGD: Focus Group Discussion; HIV: Human immunodeficiency virus; SD: Standard deviation; SSA: Sub Saharan Africa; TACAIDS: Tanzania Commission for AIDS; UNAIDS: Joint United Nations Programme on HIV/AIDS.

## Competing interests

Authors declare that there is no competing interest.

## Authors’ contributions

TK: participated in data collection, analysis, interpretation of results, and writing of the manuscript; HA: participated in data collection, interpretation of results; RK: participated in the conception and design of the study, supervised data collection, and interpretation of results; and BPM: participated in interpretation of results and re-writing of earlier version of the manuscript. All authors read the manuscript and approved it.

## Pre-publication history

The pre-publication history for this paper can be accessed here:

http://www.biomedcentral.com/1471-2458/14/630/prepub

## Supplementary Material

Additional file 1 Questionnaire for employees. Click here for file

Additional file 2 Questionnaire for employers. Click here for file

Additional file 3 Guiding questions for focus group discussions. Click here for file
