# Supplementary material for: Health and social support services to HIV/AIDS infected individuals in Tanzania: employees and employers perceptions
Source: BMC Public Health. 2014 Jun 20;14:630. doi: 10.1186/1471-2458-14-630 (PMC4074831; doi:10.1186/1471-2458-14-630)
Supplement: Additional file 1 — Questionnaire for employees. [file 1471-2458-14-630-S1.doc]

**Study on Assessment of Provision of Health and Social Support Services to HIV/AIDS Infected Employees in Arusha, Dar es Salaam and Tanga Regions in Tanzania**

# Tool 1: Employees

**A: GENERAL INFORMATION**

Questionnaire No: |__|__|__|

Interviewer’s ID No. |___|___|

Date of interview: |__|__|/|__|__|/2006

Region: __________________________

Name of workplace (Company/Firm):_____________________________________________

Nature of activity of workplace (Company/Firm):____________________________________

B: DEMOGRAPHIC CHARACTERISTICS

| **NO.** | **Questions and filters** | **Response** | **Skip to** |
| --- | --- | --- | --- |
| 1 | *Do not ask this question, circle that apply*  Sex of respondent | Male….1  Female….2 |  |
| 2 | How old are you now? | Years old|__|__| |  |
| 3 | What is your education level? | No formal education……1  Primary not finished……2  Primary finished……3  Technical/vocational education……4  Secondary education……5  College/ university……6  Other……96  (*Specify*)______________________ |  |
| 4 | What is your marital status? | Never married……1  Married……2  Widowed……3  Divorced……4  Separated……5 |  |
| 5 | Have you ever had a child? | Yes……1  No……2 |  |
| 6 | For how long have you been working at this workplace? | Years at workplace |__|__| |  |
| **C: SPECIFIC INFORMATION** | | | |
| 7 | Some employers require people to undergo HIV test before employment. Was it a requirement for your employment at this workplace? | Yes……1  No……2 | →9 |
| 8 | Being HIV+ or HIV- was it considered to be a qualification for your employment at this workplace? | Yes……1  No……2 |  |
| 9 | Do you have any member of your family or close relative who is a HIV+ or has AIDS? | Yes……1  No……2 | →11 |
| 10 | If *yes*, what support do you give him/her?  *Circle all that apply* | Treatment or health care support…1  Psychological support…2  Other……96  (*Specify*)______________________ |  |
| 11 | Is there any health or social support from your employer? | Yes……1  No……2 | →13 |
| 12 | If yes, what are they?  (a) Health support:  *Circle all that apply* | Treatment/health care…..1  Nutritional support…..2  Other……96  (*Specify*)______________________ |  |
|  | (b) **Social support:**  *Circle all that apply* | Psychosocial support …..1  Psychological support …..2  Spiritual need…..3  Home visiting…..4  Soft loans…..5  Reduced workload…..6  Other……96  (*Specify*)______________________ |  |
| 13 | Which specific support do you need from your employer?  (a) Health support:  *Circle all that apply* | Treatment/health care …..1  Nutritional support…..2  Other……96  (*Specify*)______________________ |  |
|  | (b) Social support:  *Circle all that apply* | Psychosocial support …..1  Psychological support …..2  Spiritual need…..3  Home visiting…..4  Soft loans…..5  Reduced workload…..6  Other……96  (*Specify*)______________________ |  |
| 14 | If a person knows that he/she has AIDS, do you think that he/she should keep private or tell his/her employer? | Keep private …..1  Should tell employer …..2  Don’t Know …..97 |  |
| 15 | Are there any forms of HIV/AIDS-related discrimination among infected employees at your workplace? | Yes……1  No……2 | →18 |
| 16 | If *yes*, what are they? | Eating at the same table …..1  Using the same toilet …..2  Working in the same office …..3  Promotion…..4  Further training…..5  Privilege…..6  Other……96  (*Specify*)______________________ |  |
| 17 | In your opinions, what should be done to stop/eliminate discrimination of HIV/AIDS infected employees in the workplace? |  |  |
| 18 | Has your employer ever talked about discrimination of HIV/AIDS infected employees at the workplace? | Yes …..1  No …..2  Don’t Know …..97 |  |
| 19 | Is there a specific unit/programme/club that deals with HIV/AIDS issues at your workplace? | Yes …..1  No …..2  Don’t Know …..97 | →21  →21 |
| 20 | If *yes*, have you ever attended any health education programme on HIV/AIDS care, prevention, and impact mitigation? | Yes …..1  No …..2 |  |
| 21 | How do you regard HIV/AIDS infected employees? | Like any other person …..1  Stigma …..2  Don’t Know …..97 |  |
| 22 | Have there been any complaints on stigma for people living with HIV/AIDS at the workplace? | Yes …..1  No …..2  Don’t Know …..97 | →25  →25 |
| 23 | If *yes*, who causes the situation? | Co-workers …..1  Management …..2  Customers …..3  Other……96  (*Specify*)______________________ |  |
| 24 | In your opinions, what should be done to stop/eliminate stigma on HIV/AIDS infected employees? |  |  |
| 25 | Are there any income generating activities apart from your salary which your employer has helped you to raise your income? | Yes …..1  No …..2 | →27 |
| 26 | If *yes*, what types of support? |  |  |
| 27 | Does your employer provide support to orphans of the former employees? | Yes …..1  No …..2  Don’t Know …..97 | →29  →29 |
| 28 | If *yes*, what types of support? |  |  |
| 29 | Do you agree or disagree with the idea that employers should provide health and social support to their workers who happen to be infected with HIV? | Agree…..1  Disagree…..2  Don’t Know …..97 |  |

***Thank you very much for taking your time to respond to our questions***
