# Supplementary material for: Health and social support services to HIV/AIDS infected individuals in Tanzania: employees and employers perceptions
Source: BMC Public Health. 2014 Jun 20;14:630. doi: 10.1186/1471-2458-14-630 (PMC4074831; doi:10.1186/1471-2458-14-630)
Supplement: Additional file 2 — Questionnaire for employers. [file 1471-2458-14-630-S2.doc]

**Study on Assessment of Provision of Health and Social Support Services to HIV/AIDS Infected Employees in Arusha, Dar es Salaam and Tanga Regions in Tanzania**

# Tool 2: Employers (workplace managers)

**A: GENERAL INFORMATION**

Questionnaire No: |__|__|__|

Interviewer’s ID No. |___|___|

Date of interview (dd/mm/yyyy): |__|__|/|__|__|/2006

Region: __________________________

Name of workplace (Company/Firm):_____________________________________________

Nature of activity of workplace (Company/Firm):____________________________________

Year of commencement of services/production: |__|__|__|__|

**B: SPECIFIC INFORMATION**

| **NO.** | **Questions and filters** | **Response** | **Skip to** |
| --- | --- | --- | --- |
| 1 | How many employees do you have at your workplace? | Male employees |__|__|__|__|  Female employees |__|__|__|__| |  |
| 2 | Do you currently have any employee at your workplace that is HIV/AIDS infected? That is, any employee who is living with HIV/AIDS or has any member of his/her family or close relative who is living with HIV/AIDS? | Yes…..1  No…..2  Don’t Know…..97 | →4  →4 |
| 3 | If *yes*, how many? | Employees |__|__|__|  Don’t Know…..97 |  |
| 4 | How do you normally know that your employee is HIV/AIDS-infected? That is, is living with HIV/AIDS or has a member of the family or close relative who is living with HIV/AIDS? | Hear saying……1  Prescription from a doctor……2  Unit at your workplace……4  Individual employee……5  Other……96  (*Specify*)______________________ |  |
| 5 | What do you normally do to a HIV/AIDS-infected employee?  *Circle all that apply* | Terminate from work……1  Reduce his/her workload ……2  Change work position……3  Provide treatment ……4  Leave as it is……5  Other……96  (*Specify*)______________________ |  |
| 6 | In your opinion, what is your employees’ attitude towards HIV/AIDS-infected workers? | Negative against them ……1  Positive attitude ……2  Neutral……3  Don’t Know…..97 |  |
| 7 | What are the strategies that you have adopted to ensure prevention of HIV infection at your workplace? |  |  |
| 8 | Is there any health education programme for employees and their families on matters related to HIV/AIDS? | Yes…..1  No…..2 | →13 |
| 9 | If *yes*, how often this kind of programme is provided per year? | Once per year……1  Twice per year……2  Three times per year……3  Four times per year……4  Other……96  (*Specify*)______________________ |  |
| 10 | Who normally provides this programme? | Staff within the workplace……1  Trainer from outside the workplace……2  Both 1&2……3  Other……96  (*Specify*)______________________ |  |
| 11 | Who normally participates in this training programme?  *Circle all that apply* | Managers…..1  Low level workers…..2  All employees…..3  Employees and their families…..4 |  |
| 12 | From where are these training sessions normally conducted? | Within the workplace…..1  Outside the workplace…..2  Both 1 & 2…..3 |  |
| 13 | Do you have a trained person(s) to manage HIV/AIDS prevention and follow up of infected employees at your workplace? | Yes…..1  No…..2 |  |
| 14 | Do you have any Information, Education and Communication (IEC) materials for HIV/AIDS matters at your workplace? | Yes…..1  No…..2 |  |
| 15 | Do you have any system of treatment for employees at your workplace? | Yes…..1  No…..2 | →17 |
| 16 | If *yes*, what treatment procedures for employees do you have at your workplace? |  |  |
| 17 | What measures do you have at your workplace for employees with long term illnesses? |  |  |
| 18 | In case it happens that your employee dies, what funeral services do you provide? | Provide coffin…..1  Provide transport…..2  Provide fuel…..3  Provide cash money…..4  Meet all burial costs…..5  Other……96  (*Specify*)______________________ |  |
| 19 | Do you normally provide any health support services to HIV/AIDS-infected employees at your workplace?  (*Remind the respondent about the survey’s definition of a HIV/AIDS-infected employee*) | Yes…..1  No…..2 | →21 |
| 20 | If *yes*, what types of health support services do you normally provide to HIV/AIDS infected employees at your workplace?  *Circle all that apply* | Treatment/medical care…..1  Nutritional support…..2  Other……96  (*Specify*)______________________ |  |
| 21 | Do you normally provide any social support services to HIV/AIDS infected employees at your workplace? | Yes…..1  No…..2 | →23 |
| 22 | If *yes*, what type of social support services do you normally provide to HIV/AIDS infected employees at your workplace?  *Circle all that apply* | Psychosocial support…..1  Psychological support…..2  Non-discriminatory working environment…..3  Spiritual needs…..4  Reduced workload…..5  Income generating activities…..6  Other……96  (*Specify*)______________________ |  |
| 23 | Some employers require people to undergo HIV test before employment. Is it a requirement for at your workplace? | Yes…..1  No…..2 | →25 |
| 24 | If *yes*, which level of staff at your workplace has to undergo this test? | Managerial level only…..1  Low level only…..2  All levels…..3 |  |
| 25 | In your views, why do you think some employers do not like to employ HIV/AIDS-infected people? |  |  |
| 26 | How do you normally divide roles/responsibilities between HIV/AIDS-infected and non-infected employees? | Assign different roles…..1  Less roles/responsibilities to HIV/AIDS-infected employees…..2  Equal roles and responsibilities for both…..3 |  |
| 27 | Have you ever received any complaints/conflicts from HIV/AIDS infected or non-infected employees? | Yes…..1  No…..2 | →29 |
| 28 | . If *yes*, what are these complaints/conflicts about? | Roles/responsibility conflict…..1  Recognition…..2  Respect…..3  Discrimination…..4  Sexual assault…..5  Other……96  (*Specify*)______________________ |  |
| 29 | Do you have any system of favouring HIV/AIDS infected employees at your workplace? | Yes…..1  No…..2 | →31 |
| 30 | If *yes*, what are these favours  *Circle all that apply* | Providing allowances…..1  Providing loans…..2  Project activities…..3  Promotion…..4  Other……96  (*Specify*)______________________ |  |
| 31 | If *no*, what is your opinion in regards to supporting HIV/AIDS infected employees? |  |  |

***Thank you very much for taking your time to respond to our questions***
