# Supplementary material for: Health and social support services to HIV/AIDS infected individuals in Tanzania: employees and employers perceptions
Source: BMC Public Health. 2014 Jun 20;14:630. doi: 10.1186/1471-2458-14-630 (PMC4074831; doi:10.1186/1471-2458-14-630)
Supplement: Additional file 3 — Guiding questions for focus group discussions. [file 1471-2458-14-630-S3.doc]

**Study on Assessment of Provision of Health and Social Support Services to HIV/AIDS Infected Employees in Arusha, Dar es Salaam and Tanga Regions in Tanzania**

# Tool 3: Focus Group Discussion

**A: GENERAL INFORMATION**

First Facilitator’s ID No.: |___|___|

Second Facilitator’s ID No.: |___|___|

Note Taker’s ID No.: |___|___|

Date of Discussion (dd/mm/yyyy): |__|__|/|__|__|/2006

Number of Participants: |___|___|

Region: __________________________

Name of workplace (Company/Firm):_____________________________________________

Nature of activity of workplace (Company/Firm):____________________________________

**B: DISCUSSION GUIDE QUESTIONS**

1. What measures are taken by your employer to hand cases of employees with prolonged illnesses?

2. How does your employer divide roles/responsibilities among HIV/AIDS infected and non-infected employees at your workplace?

3. Does your employer provide any health or social support to HIV/AIDS infected employees? What types of health and social support are provided by your employer?

4. Are there any support given by the employer to orphans or widows of former employees?

5. Are there any forms of HIV/AIDS-related stigma and discrimination at your workplace?
